# Temporal discounting for self and friends in adolescence: A fMRI study

**DOI:** 10.1016/j.dcn.2023.101204

**Published:** 2023-01-27

**Authors:** Suzanne van de Groep, Sophie W. Sweijen, Erik de Water, Eveline A. Crone

**Affiliations:** a Erasmus SYNC Lab, the Netherlands; b Erasmus School of Social and Behavioral Sciences, Erasmus University Rotterdam, the Netherlands; c Brain and Development Research Center, the Netherlands; d Leiden Institute for Brain and Cognition, the Netherlands; e Great Lakes Neurobehavioral Center, Edina, MN, United States

**Keywords:** Temporal discounting, Impulsivity, Social, Adolescence, Reward, Friend

## Abstract

Adolescence is characterized by impulsivity but also by increased importance of friendships. This study took the novel perspective of testing temporal discounting in a fMRI task where choices could affect outcomes for 96 adolescents (aged 10–20-years) themselves and their best friend. Decisions either benefitted themselves (i.e., the Self Immediate – Self Delay’ condition) or their friend (i.e., ‘Friend Immediate – Friend Delay’ condition); or juxtaposed rewards for themselves and their friends (i.e., the ‘Self Immediate – Friend Delay’ or ‘Friend Immediate – Self Delay’ conditions). We observed that younger adolescents were more impulsive; and all participants were more impulsive when this was associated with an immediate benefit for friends. Individual differences analyses revealed increased activity in the subgenual anterior cingulate cortex extending in the ventral striatum for immediate relative to delayed reward choices for self. Temporal choices were associated with activity in the prefrontal cortex, parietal cortex, insula, and ventral striatum, but only activity in the right inferior parietal lobe was associated with age. Finally, temporal delay choices for friends relative to self were associated with increased activity in the temporo-parietal junction and precuneus. Overall, this study shows a unique role of the social context in adolescents’ temporal decision making.

## Highlights

• Adolescents’ temporal discounting choices are impacted by the social context (i.e., outcomes for self versus friend).
• Adolescents were more impulsive when this was associated with an immediate benefit for friends.
• Delay choices for friends relative to self were associated with TPJ and precuneus activity, possibly reflecting mentalizing.
• Overall, findings highlight behavioral and neural mechanisms underlying adolescents’ temporal choices for self and friends.

## 1 Introduction

Adolescence is a period in life in which individuals learn to balance different goals, such as those related to immediate versus future goals (e.g., hanging out with friends vs. studying for a test), and those related to goals for self or others (e.g., spending some time relaxing vs. helping a friend with homework; Crone and Fuligni, 2020; Steinberg et al., 2009). Balancing such considerations is needed to successfully grow up into an adult member of society. The ability to successfully act on future instead of immediate goals has been associated with higher grades and less substance use, risky sexual behavior, and gambling (Lee et al., 2012; Reynolds, 2006). Although there is converging evidence that balancing both temporal personal goals as well as social goals is needed to successfully grow up (Crone and Fuligni, 2020), it remains largely unknown how adolescents’ make temporal decisions for others, especially when they have to balance this with rewarding outcomes for themselves.

The ability to successfully act on future instead of immediate goals is reflected in adolescents’ temporal discounting, which refers to their tendency to perceive a desired result in the future as less desirable than in the present (Matta et al., 2012). The development of temporal discounting has often been studied using paradigms in which individuals have to decide between an immediate small reward, and a larger future reward (Critchfield and Kollins, 2001, de Water et al., 2017, Steinberg et al., 2009). Choosing an immediate over delayed reward is often referred to as impulsivity, although it should be noted that impulsivity can be seen a broader multi-dimensional construct capturing the extent to which someone acts on a whim or without much forethought (Reynolds, 2006). Temporal discounting studies mostly show that younger adolescents show steeper discounting rates (i.e., more impulsivity) compared to older adolescents – that is, they prefer immediate over larger delayed reward as delays preceding the larger reward increase. Temporal discounting continues to develop until late adolescence or early adulthood (de Water et al., 2014; Lee et al., 2013; Olson et al., 2007; Scheres et al., 2006; Steinberg et al., 2009; Van Den Bos et al., 2015; Yu et al., 2021). Most studies suggest linear patterns, although some studies report either a quadratic pattern (Achterberg et al., 2016, Scheres et al., 2014) or no age differences (Audrain-McGovern et al., 2009, de Water et al., 2017), showing that age effects are dependent on the specific samples (e.g., sample sizes and ages) and context (e.g., used reward and delay magnitude).

Recently, it has been suggested that adolescents can flexibly exert more self-control in situations where they are highly motivated to do so, such as non-hypothetical tasks and tasks with high monetary payoffs (Crone and Dahl, 2012, Scheres et al., 2014). However, adolescents often do not only make decisions between immediate and future personal outcomes, but also between outcomes for self and others. Studies have shown that adolescents differentiate between the subjective pleasure they experience when winning for self and friends compared to unknown others (Braams et al., 2014), demonstrating that the social context plays an important role in adolescent decision making (van Hoorn et al., 2019). Overall, this suggests that adolescents may choose a delayed over an immediate option when the social stakes are high.

Neuroimaging studies have allowed for a more mechanistic understanding of developmental change in temporal discounting using task-based functional magnetic resonance imaging (fMRI). These fMRI studies have elucidated two brain systems that are involved in temporal discounting: one associated with impulsivity and reward seeking (localized mainly in subcortical, limbic areas) which develops relatively early (i.e., this system is in place and highly active during adolescence), and one associated with more deliberative and abstract reasoning, as well as planning, localized in the prefrontal cortical regions of the brain, which develops relatively late (i.e., into the late 20 s; Steinberg et al., 2009). This neural imbalance (Casey et al., 2008, Crone and Dahl, 2012, Galvan et al., 2007) may predispose adolescents towards relatively impulsive decisions, such as selecting immediate over delayed rewards (de Water et al., 2017, Galvan et al., 2007). Prior studies that used temporal discounting paradigms in adolescence confirmed that these cortical and subcortical brain regions are involved when comparing temporal discounting choices to non-temporal discounting (i.e., control) choices (de Water et al., 2017, Stanger et al., 2013). In addition, one study in adolescents showed that delayed compared to immediate reward choices are associated with activation in the superior parietal cortex, a region that plays a role in cognitive control, conflict monitoring, and attention (de Water et al., 2017).

Next, there is evidence that individual differences may play a larger role in explaining neural activation related to immediate vs. delayed rewards in adolescence than normative age effects. Relatively impulsive individuals (i.e., those that more often selected an immediate over a delayed reward) engage various frontal and parietal (i.e., control) regions more strongly during delayed reward choices, and the ventral striatum and mPFC (e.g., reward-related regions) during immediate reward choices, suggesting that they have to overcome high reward sensitivity by exerting more control (Albrecht et al., 2011, de Water et al., 2017, Stanger et al., 2013). Overall, these studies show an important role for control-related neural circuitry and reward-related neural circuitry in temporal discounting choices in adolescence.

When considering the outcomes of others, individuals need to consider the thoughts and beliefs of others, or whether their decision affects outcomes for both themselves and others (Blakemore, 2008, Crone and Fuligni, 2020). Mentalizing abilities (i.e., the ability to understand one’s own and other’s mental states and the associated behavior; Blakemore, 2008; Steinberg and Morris, 2000) improve with age across adolescence (Poznyak et al., 2019, van den Bos et al., 2011), possibly increasing the likelihood that adolescents show other-benefitting behaviors, such as giving and helping (Crone et al., 2022). Indeed, studies have shown that adolescents are more generous to unknown others when they are better at perspective-taking (van de Groep et al., 2020). When thinking about outcomes for self and others, a special role is reserved for social cognition or mentalizing regions, which is a network of brain regions involved in understanding and interacting with others, such as the medial prefrontal cortex (i.e., mPFC), temporal-parietal junction (i.e., TPJ), precuneus, and superior temporal sulcus (i.e., STS; (Blakemore, 2008, Crone et al., 2022, Crone and Dahl, 2012, Crone and Fuligni, 2020, van Hoorn et al., 2019). Interestingly, some of these regions, such as the TPJ, seem particularly sensitive to decisions for close others, such as friends (van de Groep et al., 2022), suggesting that these regions also play an important role in integrating social contextual information into decisions (Crone et al., 2022, Crone and Fuligni, 2020). Overall, these findings suggest that temporal decisions contrasting self and others are likely to call upon social cognition-implicated regions as they require adolescents to consider their own and others’ perspectives.

Taken together, although adolescents’ behavior depends both on temporal as well as social goals, and these processes are likely to co-occur in everyday decision making, few studies to date have connected them (Albrecht et al., 2011, Crone et al., 2008, Mahy et al., 2020, Neff and Macaskill, 2021, Ziegler and Tunney, 2012). Earlier studies using temporal discounting paradigms have elucidated that the ability to self-regulate in temporal decisions is predictive of resisting temptation to achieve long-term goals later in life (Casey et al., 2011). In adolescents’ real-life decisions, the ability to self-regulate and select a delayed reward are likely to be influenced by adolescents’ sensitivity to rewarding outcomes for self and others and may therefore not only impact long-term personal goal achievement, but also reaching long-term interpersonal goals, such as building long-lasting, meaningful social relationships with others (Crone et al., 2022, Crone and Fuligni, 2020). As such, testing how temporal discounting interacts with the individually varying balance of valuing outcomes for self and others is likely to give additional insight into the behavioral and neural mechanisms underlying adolescents’ temporal decision making for self and friends, which may impact the balance between personal and interpersonal goal achievement, and thus social adjustment, later in life.

There have not been many studies that have examined social temporal decision making, but the few that have in adults and children suggest that individuals are generally better at selecting a larger, delayed rewards for others than for themselves (Albrecht et al., 2011, Mahy et al., 2020), and one study in 13–16 year-old adolescent boys showed that this is particularly true when the other shows low sensation seeking (Crone et al., 2008). There is also evidence that this other-over-self preference may be specific to certain developmental periods but this has not yet been investigated in adolescents (Albrecht et al., 2011, Mahy et al., 2020). To date, only one fMRI study, which included 28 young adult participants, has focused on temporal discounting for self and others (Albrecht et al., 2011). This study, in which the other was a stranger, showed an interaction between temporal discounting and receiver (self. vs. other), such that participants showed the highest activation in the posterior anterior cingulate cortex (ACC), anterior mPFC, and ventral striatum for immediate rewards for the self. This suggests that immediate temporal decisions for the self may be more salient and rewarding compared to temporal decisions for others (Albrecht et al., 2011). However, as these results were obtained in young adults, it remains unclear whether adolescents discount delayed rewards similarly for themselves and others, particularly when outcomes for self and other are juxtaposed. The current study set out to examine whether social (i.e., self vs. friend) considerations within the context of temporal discounting call upon separate or overlapping neural regions in adolescent development, with preliminary studies suggesting that these processes engage separable brain regions (Albrecht et al., 2011, Ersner-Hershfield et al., 2009).

The aim of the current study was to investigate temporal discounting for self and friends, both in terms of behavior and associated neural activity, and age patterns across adolescence. We selected the friend as a target because adolescence is a phase in life marked by a social reorientation towards and increased importance of friends (Berndt, 1992). This is underscored by prior studies that demonstrated an adolescence-specific peak in sensitivity to rewards for stable best friends (Schreuders et al., 2021) and higher levels of giving, trust, and reciprocity towards friends compared to unknown others in adolescence (Güroğlu et al., 2014, van de Groep et al., 2020, van de Groep et al., 2022). For the current study, we designed a temporal discounting task in which adolescents had to decide between a small, immediate reward, and a large, delayed reward, in four conditions, whilst they were undergoing functional magnetic resonance imaging (fMRI). These decisions either solely affected themselves or their friend (i.e., the ‘Self Immediate – Self Delay’ and ‘Friend Immediate – Friend Delay’ conditions) or represented a weigh-off between outcomes for themselves and their friends (i.e., the ‘Self Immediate – Friend Delay’ or ‘Friend Immediate – Self Delay’ conditions). This design allowed us to disentangle the behavioral and neural patterns associated with these different conditions within individuals, and to map possible age-related differences. As such, the temporal discounting task allowed for detection of separable or overlapping neural correlates across conditions.

We expected that adolescents would more often select the delayed for friends than for themselves (Mahy et al., 2020, Neff and Macaskill, 2021). We exploratorily examined differences between the conditions where choices for self and others were pitted against each other. Second, for decisions that solely involved the self, we expected an age-related decrease in temporal discounting, reflected in higher area under the curve (AUC) values for older adolescents (de Water et al., 2014, Olson et al., 2007, Scheres et al., 2006, Steinberg et al., 2009). Third, we explored whether there was an interaction between age and task conditions to unravel whether the development of discounting is dependent on the social context (Albrecht et al., 2011). Fourth, we expected activation in prefrontal and subcortical regions for temporal discounting (averaged over conditions) versus control trials (de Water et al., 2017, Steinberg et al., 2009). Fifth, we expected that neural activation related to immediate and delayed rewards would depend on the specific task condition and AUC values (de Water et al., 2017). We tested whether relatively immediate reward decisions for the self are associated with ventral striatal activation (Albrecht et al., 2011), and decisions that benefit the other over the self with activation in social brain regions including the TPJ, precuneus, and mPFC (Crone et al., 2022). Finally, we also explored neural age effects associated with temporal discounting (i.e., TD) versus control choices, immediate versus delayed rewards, and the four task conditions.

## 2 Method

### 2.1 Participants

Participants in this study were 108 adolescents (66 females), between the ages of 9 – 20 (*M*_age_ = 15.95, *SD*_age_ = 2.73, age range 9.69 – 20.21 years). Participants were invited to participate in the current study as part of the second measurement wave of a three-wave longitudinal project on the development of prosocial behavior in adolescence called ‘Brainlinks’ (Crone et al., 2022). In total, 12 participants were excluded because they showed excessive (> 3 mm) head movement during the task (see MRI data acquisition). The final sample consisted of 96 participants (58 females) (*M*_age_ = 16.44, *SD*_age_ = 2.43, age range 10.16 – 20.21 years). Approximation of IQ was determined by two subscales (i.e., similarities and block design) of the Wechsler Intelligence Scale for Children (WISC) for participants aged 16 and younger and Wechsler Adult Intelligence Scale (WAIS) for participants aged 17 or older. This data was available for 82 of the 96 participants in the final sample. Estimated IQ for these 82 participants fell within the normal range (*M* = 107.53, *SD* = 11.34). Participants were mainly from Dutch origin and the social economic status as assessed by parental educational level was middle to high.

Participants were contacted by telephone to assess their willingness to participate, for which they had previously given permission. Participants provided written informed consent at the start of the lab visit, and for minors (i.e., ages 15 and younger) both parents also provided written informed consent. Participants had normal or corrected-to-normal vision, and no diagnosed intellectual disability. Participants were screened for neurological and psychiatric disorders and MRI contraindications via a private telephone conversation. This study was approved by the local medical ethical committee. Participants received 30 euros (< 12 years) or 40 euros (ages ≥ 12 years) and small presents for their participation, plus additional payout for the temporal discounting fMRI task and other tasks that were performed as part of the larger study protocol.

### 2.2 Materials: temporal discounting task for self and friend

To assess the neural correlates of temporal discounting for self and friend, we developed a modified version of a fMRI temporal discounting task with monetary rewards in the MRI scanner (de Water et al., 2017). In keeping with prior neuroimaging studies on temporal discounting, the task was potentially real, such that one choice was randomly selected by the computer for payout. If the participant chose an immediate reward, they received the respective monetary reward at the end of the lab visit (either for themselves or for their friend, in which case they were asked to give the money to their friend). If the participant chose a delayed reward, the money was transferred to their bank account after the corresponding delay. In cases where the delayed reward represented a win for the friend, participants were asked to give this money to their friend. As such, all choices had real monetary payout consequences (de Water et al., 2017, Scheres et al., 2013).

In the current task with an event-related design, participants made temporal discounting choices concerning themselves and their best friend in four conditions: they either decided 1) between a small reward for the self now, and a small reward for the self later (the ‘Self Immediate – Self Delay’ condition), 2) between a small reward for the friend now, and a large reward for the friend later (the ‘Friend Immediate – Friend Delay’ condition), 3) between a small reward for the self now, and a large reward for the friend later (the ‘Self Immediate – Friend Delay’ condition), or 4) between a small reward for the friend now, and a large reward for the self later (the ‘Friend Immediate – Self Delay’ condition). For each of these conditions, there were 40 trials in total, and we varied both the immediate reward magnitude (2, 4, 6, or 8 euros) and the delay in days (2, 14, 30, 90, and 180 days). The delayed reward was always 10 euros. Each immediate reward was presented 40 times, and each delay variation was presented 32 times. See Supplement 1 for the number of times each combination of task conditions, immediate rewards, and delays was displayed. In addition, there was a control condition (20 trials), where participants selected the larger of two circles. This condition allowed us to compare neural activity in response to TD choices and choices that required no consideration of reward amounts or delays, but only a perceptual decision and motor response (de Water et al., 2017). As such, the task consisted of 180 trials in total, which were divided over three runs. Participants completed 30 practice trials before entering the scanner. The scanner task lasted approximately 23 min (i.e., 7.5 min per run).

The task was presented in the MRI scanner using E-Prime version 2 (Schneider et al., 2012). Trials started with a jittered fixation cross for 1000–3000 ms, which was optimized using OptSeq (Dale, 1999), after which participants indicated their preference by pressing a button on a button response box with their right index finger (left option) or right middle finger (right option). Participants were given 4000 ms to select their preferred option, after which their choice was highlighted for 1000 ms via a white frame around the selected choice. If participants did not press in time, a ‘too late’ screen was shown for 1000 ms and these trials were excluded from analysis. The position of the options on the screen (i.e., the immediate and delayed reward) was counterbalanced across participants.

To assess temporal discounting, we calculated participants’ subjective value (SV) of each reward for each of the delays (Mies et al., 2018). The subjective value was calculated by taking the number of choices for the delayed reward per delay (2, 14, 30, 90, and 180 days) and dividing this by the total number of choices per delay. This number was then multiplied by 8 (i.e., the range of plausible SVs in our task), after which 1 (the lowest plausible SV in our task) was added, following the method of de Water et al. (2022). These SVs were then used to calculate the area under the curve (AUC) using the procedure described by Myerson et al. (2001). AUC is a common measure of temporal discounting, where smaller values indicate an increased preference for immediate rewards (Myerson et al., 2001). AUC ranges between 0 and 1, where smaller values indicate an increased preference for immediate rewards. To assess the effects of task condition on temporal discounting, we performed a repeated measures ANOVA with AUC as dependent variable and task condition as within-subjects factor. To examine age effects, we added age (linear and quadratic) to the RM ANOVA in a hierarchical manner. Analyses examining behavioral and age effects for specific delays and immediate reward magnitudes are described in Supplement 2.

### 2.3 Procedure

As the current study was part of the second measurement wave of a longitudinal study, participants completed additional tasks and questionnaires apart from the temporal discounting fMRI scan. At the start of the lab visit, participants were instructed about the visit, MRI scanner, and measurements. The MRI session consisted of a structural scan, resting state scan, two fMRI tasks (one of which was the temporal discounting task), and a diffusion tensor imaging scan. Finally, participants filled out questionnaires and performed tasks on social decision making outside the MRI scanner.

### 2.4 MRI data acquisition

MRI scans were acquired using a 3 T MRI scanner (Philips Achieva TX, Erlangen, Germany) with a standard whole-head coil. Stimuli were displayed on a screen, which participants could see through a mirror attached to the head coil. For anatomical reference, we collected a high-resolution 3D T1-weighted anatomical image (isotropic voxel size 1.1 mm3, RT =7.9 ms, TE = 3.5 ms, flip angle = 8º, FOV = 250 ×196 x 171 mm, duration = 04:12 s). T1 stabilization dummy scans were automatically discarded by the scanner. Functional scans were acquired during 3 runs, which consisted of 196 dynamic scans each. We collected T2 * weighted gradient echo planar images (EPI; TR = 2.2 s, TE = 30 ms, flip angle 80º, sequential acquisition: 38 slices, voxel size = 2.75 ×2.75 ×2.75 mm, 80 ×77 matrix, field of view (VOF) = 220 × 220 x 115 mm). Before the start of each functional run, 5 dummy scans were made. All scans were acquired using a fast field echo pulse sequence. To avoid head motion, foam inserts were used at both sides of the head when possible. Before exclusion of participants who showed excessive head motion (*N* = 108), movement was as follows: movement range:.00 – 9.23 mm, *M* = 0.12, *SD* = 0.15. Movement for the final sample (*N* = 96) was as follows:.00 – 2.75, *M* = 0.09, *SD* = 0.09. Movement in the final sample was negatively correlated with age, *r* = −0.32, *p* < .001, such that younger adolescents moved more. Therefore, to prevent loss of power in each cell of this study’s design and in detecting age effects, we used a exclusion rule of > 3 mm in any direction and added motion parameters to the design rather than scrubbing (Achterberg et al., 2018). The full information on movement parameters in the current sample is presented in Supplement 3.

### 2.5 MRI data analysis

#### 2.5.1 Preprocessing

MRI data analysis was executed using SPM8 (Welcome Department of Cognitive Neurology, London, United Kingdom). Functional images were preprocessed using various steps: realignment, slice-time correction, spatial normalization using segmentation parameters, and spatial smoothing with a 6-mm FWHM isotropic Gaussian Kernel. A 12-parameter affine transform with a nonlinear transformation was used for the normalization algorithm, which involved cosine basis functions and resampled the volumes to 3-mm cubic voxels. Templates were based on MNI-305 stereotaxic space. All functional scans were corrected for excessive head motion (6 parameters).

**General linear model.** For the first-level individual analyses, we used the general linear model in SPM8. The fMRI time series were modeled as a series of zero duration events time-locked to stimulus onset and convolved with the hemodynamic response function (HRF). We used the modelled events (‘Self – Self Immediate’, ‘Self – Self Delayed’, ‘Friend - Friend Immediate’, ‘Friend - Friend Delayed’, ‘Self – Friend Immediate’, ‘Self – Friend Delayed’, ‘Friend – Self Immediate’, ‘Friend – Self Delayed’, and ‘Control’) as regressors in a general linear model, alongside motion regressors and a high pass filter of 12 Hz. Trials on which participants failed to respond were modelled separately as covariate of no interest and were excluded from analyses. In addition, we included six motion parameters as nuisance regressors. The (least square) parameter estimates (beta-weights) of the best-fitting canonical HRF for each condition were used in pairwise contrasts. These pairwise contrasts resulted in subject-specific contrast images, which were submitted to second-level group analyses.

#### 2.5.2 Whole brain analyses

**Fig. 1:**
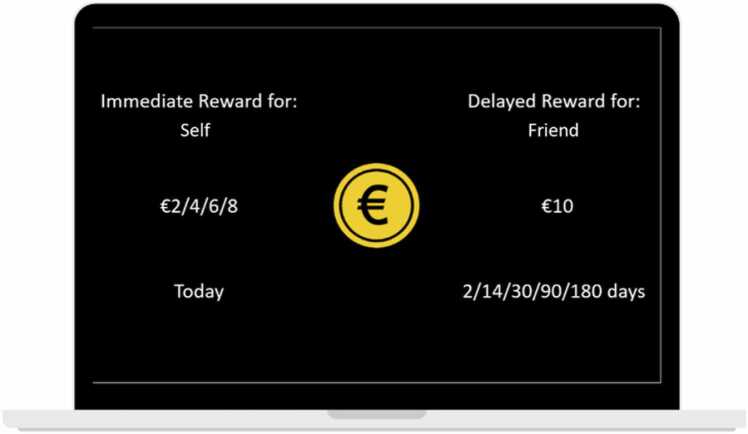
Visual display of the temporal discounting task. The task consisted of four conditions, in which participants had to choose between 1) an immediate and delayed reward for the friend, 2) an immediate and delayed reward for the friend, 3) an immediate reward for the self and a delayed reward for the friend, and 4) an immediate reward for the friend and a delayed reward for the self. Note that the third condition is displayed here. Both the immediate reward magnitude (2, 4, 5, or 8 euros) and the delay in days (2, 14, 30, 90, and 180 days) varied across trials. The delayed reward was always 10 euros. The position of the immediate and delayed reward (i.e., left or right) was counterbalanced across participants.

To examine neural activation associated with average temporal discounting choices, we contrasted TD choices with the control condition of the task and contrasted delayed to immediate choices and vice versa. To compare TD choices for the self to TD choices that also incorporated the friend, we compared the ‘Self Immediate – Self Delayed’ contrast to the contrasts ‘Friend Immediate – Friend Delayed’, ‘Self Immediate – Friend Delayed’, and ‘Friend Delayed – Self Immediate’. Finally, we examined the extent to which activity was modulated by participants’ AUC for each of these four task conditions by performing whole brain multiple regressions. To explore age effects, linear age and quadratic age were added to the whole-brain contrasts ‘Temporal Discounting vs. Control’, ‘Delay vs. Immediate’, ‘Immediate vs. Delay’, ‘Self Immediate – Self Delayed’, ‘Friend Immediate – Friend Delayed’, ‘Self Immediate – Friend Delayed’, and ‘Friend Delayed – Self Immediate’. Note that not all contrasts could be computed for all participants, as some individuals did not select certain choices. Therefore, the number of participants for which the contrast could be computed is reported in the results section. In cases where we further wanted to inspect parameter estimates related to activation in a certain cluster, we used the Marsbar toolbox in SPM8. Fig. 1.

## 3 Results

### 3.1 Behavioral results

#### 3.1.1 Temporal discounting differences between task conditions

**Fig. 2:**
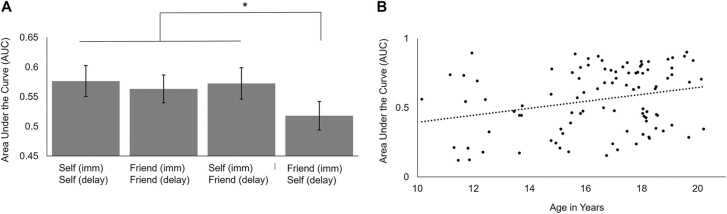
(A) Area under the curve (AUC) for each of the four task conditions. * denotes p-values ≤ 0.010. (B) Area under the curve (AUC) is higher in older adolescents, suggesting that older adolescents are less impulsive across task conditions.

To address the question whether AUC values differed for self and friends, a repeated measures ANOVA was performed with four conditions. Participants had an average AUC score of .56 (*SD* =0.23). The AUC scores differed significantly between the four task conditions, *F*(2.04, 193.32) = 6.75, *p* < .001, *η*²_p_ = .07. Pairwise comparisons using Bonferroni corrections revealed that participants had an increased preference for immediate rewards in the ‘Friend Now – Self Delay’ condition (*M* =0.52, *SD* =0.24) compared to the ‘Self Immediate – Self Delay’ (*M =.*58, *SD* =0.25, *p* = .002), ‘Friend Immediate – Friend Delay’ (*M* =0.56, *SD* =0.23, *p* = .002), and ‘Self Immediate – Friend Delay’ (*M* =0.57, *SD* =0.26, *p* = .050) conditions, see Fig. 2A. The latter three conditions did not differ significantly from each other.

Next, we addressed the question whether AUC values differed across age. Adding linear age to the analysis revealed a main effect of age; AUC was higher in older adolescents, showing that older adolescents made fewer immediate reward choices (Fig. 2B), *F*(1, 94) = 7.20, *p* = .009, *η*²_p_ = .07. There was no interaction between age and task condition. Adding quadratic age to the analysis did not reveal any quadratic age effects.

### 3.2 fMRI results

#### 3.2.1 TD vs. Control

**Fig. 3:**
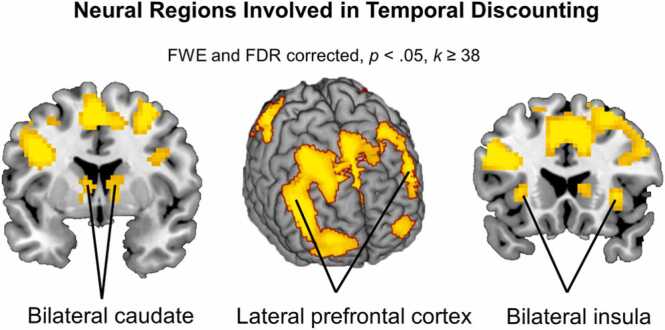
Brain areas that showed increased activation for the t-test ‘Temporal Discounting vs. Control’. Results are displayed FWE and FDR cluster correction of p < .050.

Before examining effects of task conditions, we first examined the brain regions involved in temporal discounting choices generally. To examine neural activation patterns for temporal discounting versus control trials, we performed a t-test. As expected, the contrast ‘TD vs. control’ (FWE and FDR corrected, *p* < .050; *k* ≥ 38, *N* = 84), revealed increased activation in several regions including the bilateral caudate, bilateral lateral PFC, and bilateral insula, *t*’s ≥ 6.49, p's ≤ 0.001, see Table 1 and Fig. 3A. Results regarding the reverse contrast (‘Control vs. TD’) can be found in Supplement 4.

**Table 1:** MNI coordinates of local maxima activated for the t-test for delay vs. control choices. Results were calculated using FWE and FDR cluster correction (*p≤*.050).

Area of activation	MNI Coordinates				Test statistic	Cluster Size
	x	y	z		*t*	
*T-test Delay* vs. *Control choices*						
Left Inferior Occipital	-24	-94	-8		21.19	4888
Left Supplemental Motor Area (i.e., right LPFC)	-3	11	49		12.77	1730
Right Hippocampus	24	-28	-5		9.62	60
Left Frontal Inferior Triangularis (i.e., left LPFC)	-39	17	25		9.60	689
Left Temporal Middle	-51	-37	4		8.55	82
Right Caudate	9	-16	7		7.71	316
Left Caudate	-9	-13	10		7.31	186
Right Insula	33	20	1		7.23	51
Left Insula	-30	23	1		6.63	38
Left Frontal Middle	-30	56	7		6.49	76

#### 3.2.2 Delay vs immediate and immediate vs “delay

Next, we compared brain activation for different types of choices (*N* = 85). The contrasts ‘Delay vs. Immediate’ and ‘Immediate vs. Delay’, averaged over the task conditions, did not reveal any significant clusters of activation.

#### 3.2.3 Comparison of self- and other-related task conditions

**Fig. 4:**
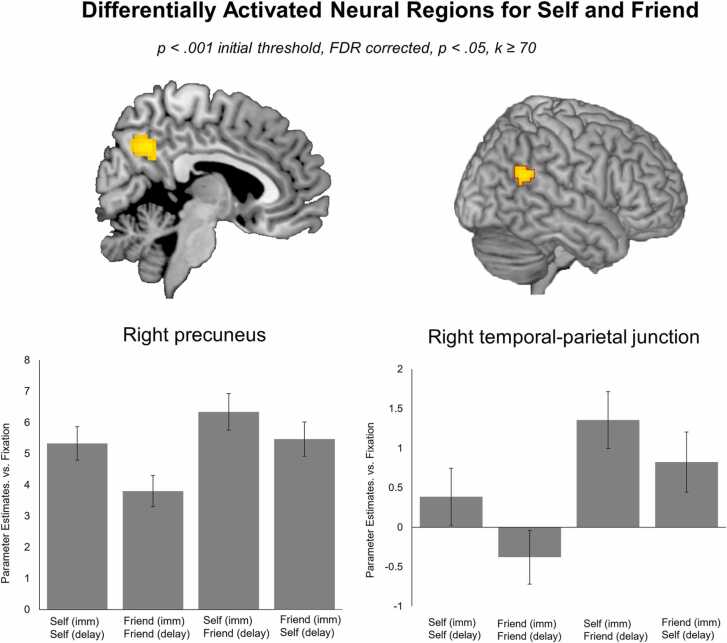
Brain areas that showed increased activation for the t-test ‘Self Immediate – Friend Delay’ vs. ‘Self Immediate – Self Delay’: the right temporoparietal junction (TPJ) and right precuneus. Results are displayed with a primary voxel-wise threshold of p < .001 (uncorrected) and FDR cluster correction of p < .050.

To compare the neural activation associated with the social vs. non-social task conditions, we performed a whole brain ANOVA with all four of the task conditions (*N*’s 89–96 per cell). To get insight into the neural correlates of the self-related versus the social conditions (i.e., the conditions that involved the friend), we specifically contrasted the three social conditions (‘Friend Immediate – Friend Delayed’, ‘Self Immediate – Friend Delayed’, and ‘Friend Delayed – Self Immediate’) to the ‘Self Immediate – Self Delayed’ condition. Only the comparison of the ‘Self Immediate – Friend Delayed’ to the ‘Self Immediate – Self Delayed’ condition, revealed increased activation in the right precuneus and right temporal-parietal junction, *t*’s ≥ 3.91, p's ≤ 0.001, see Table 2, Fig. 4. No significant clusters were observed for the other comparisons.

**Table 2:** MNI coordinates of local maxima activated for the t-test for ‘Friend Immediate – Self Delay’ versus ‘Self Immediate – Self Delay’. Results were calculated using a primary voxel-wise threshold of *p* < .001 (uncorrected), with a cluster corrected threshold of *p* < .050 FDR corrected.

Area of activation	MNI Coordinates				Test statistic	Cluster Size
	x	y	z		*t*	
*T-test ‘Friend Immediate – Self Delay’* versus *‘Self Immediate – Self Delay’*						
Right precuneus	6	-58	37		4.09	91
Righ temporal superior (i.e., right TPJ).	51	-52	22		3.91	70

Note: Names were based on the aal toolbox in SPM. For functional regions discussed throughout the paper, both the aal label and functional label (between brackets) are displayed. See *https://neurovault.org/collections/FZTVRFUL/* for a full, unthresholded overview of activation.

#### 3.2.4 Whole brain AUC effects

**Fig. 5:**
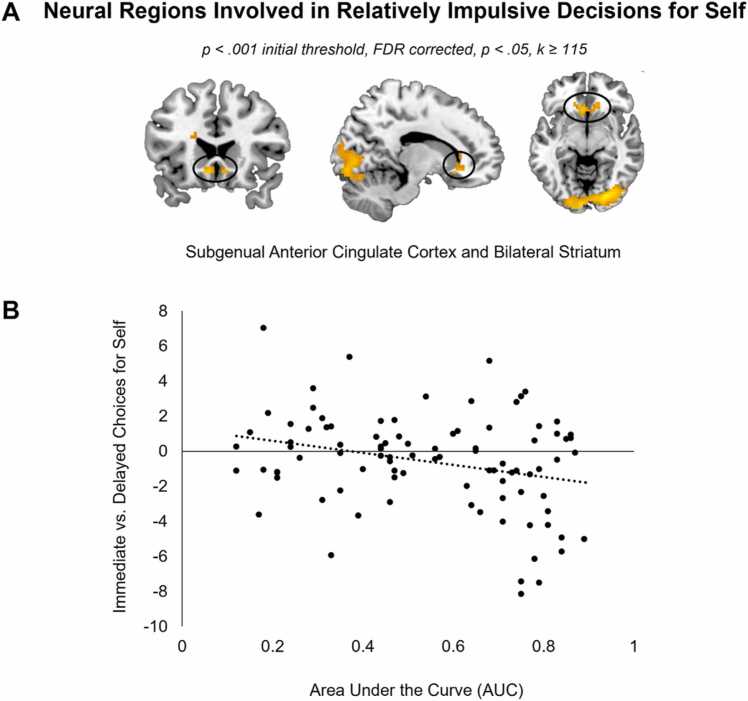
(A) Brain areas that showed increased activation for the whole brain regression investigating AUC-related modulation for ‘Self Immediate – Self Delay’. Results are displayed with a primary voxel-wise threshold of p < .001 (uncorrected) and FDR cluster correction of p < .050. (B) Association between AUC and neural activation in the cluster that includes the left and right striatum. Relatively impulsive adolescents (i.e., those with lower AUC scores) showed higher activation in this cluster.

To examine whether there were brain regions in which activity was modulated by participants’ AUC for the four conditions ‘Self Immediate – Self Delayed’, ‘Friend Immediate – Friend Delayed’, ‘Self Immediate – Friend Delayed’, and ‘Friend Delayed – Self Immediate’, we performed multiple regression analyses for each contrast (*N’s=*91, 92, 88, 92, respectively). Only the analysis comparing immediate versus delayed choices for the self revealed a significant activation cluster that included the left and right striatum, *t*= 4.94, p's ≤ 0.001, see Table 3 and Fig. 5A. As can be seen in Fig. 5A, this cluster was on the intersection of multiple regions, including the subgenual anterior cingulate cortex (ACC) and the left and right striatum. To further inspect this effect, we extracted activation for this cluster. As can be seen in Fig. 5B, adolescents who more often chose the immediate reward (i.e., had a lower AUC) showed higher activation in this subgenual ACC/striatal cluster. The other contrasts revealed no significant clusters of activation.

**Table 3:** MNI coordinates of local maxima activated for the multiple regression comparing immediate to delayed choices for the self depending on individual AUC values. Results were calculated using a primary voxel-wise threshold of *p* < .001 (uncorrected), with a cluster corrected threshold of *p* < .050 FDR corrected.

Area of activation	MNI Coordinates				Test statistic	Cluster Size
	x	y	z		*t*	
*Multiple regression for ‘Self Immediate – Self Delay’* *– clusters that regressed with AUC values*						
Right Lingual	24	-82	-8		5.85	1267
Left Olfactory (i.e., includes the left and right striatum)	-6	23	-11		4.94	135
Left Frontal Inferior Triangularis	-33	26	19		4.48	115

Note: Names were based on the aal toolbox in SPM. For functional regions discussed throughout the paper, both the aal label and functional label (between brackets) are displayed. See *https://neurovault.org/collections/FZTVRFUL/* for a full, unthresholded overview of activation.

#### 3.2.5 Whole brain age effects

**Fig. 6:**
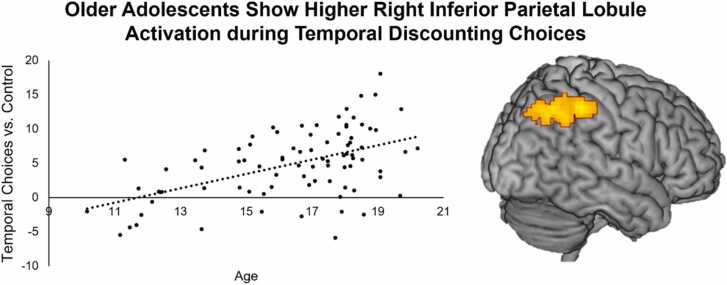
Older adolescents show higher activation in the right inferior parietal lobule for the whole brain contrast ‘temporal discounting versus control’.

Adding linear age to a whole brain multiple regression for the comparison ‘TD vs. Control’ (*N* = 84) revealed a significant cluster of activation in the right inferior parietal lobule (IPL), see Fig. 6, Table 4A. Activation in this cluster was extracted to further inspect this effect, revealing that older adolescents showed higher activation in the right IPL for temporal discounting versus control choices compared to younger adolescents. A whole brain multiple regression analysis for the comparison ‘TD vs. control’ with quadratic age (*N* = 84) revealed a cluster of activation in the superior occipital lobe, see Supplement 4. There were no linear or quadratic whole brain age effects for each of the following comparisons: ‘Temporal discounting – Control Choices’, ‘Immediate vs. Delayed (average)’, ‘Delayed vs. Immediate (average)’, ‘Self Immediate – Self Delayed’, ‘Friend Immediate – Friend Delayed’, ‘Self Immediate – Friend Delayed’, and ‘Friend Delayed – Self Immediate’.

**Table 4:** MNI coordinates of local maxima activated for the multiple regression comparing temporal discounting to control trials depending on linear age. Results were calculated using a primary voxel-wise threshold of *p* < .001 (uncorrected), with a cluster corrected threshold of *p* < .050 FDR corrected.

Area of activation	MNI Coordinates			Test statistic		Cluster Size
	x	y	z	*t*		
*Multiple regression for ‘Temporal Discounting* vs. *Control Trials’* *– clusters that regressed with linear age*						
Right Parietal Inferior (i.e., right IPL)	45	-40	49	4.78	257	

Note: Names were based on the aal toolbox in SPM. For functional regions discussed throughout the paper, both the aal label and functional label (between brackets) are displayed. See *https://neurovault.org/collections/FZTVRFUL/* for a full, unthresholded overview of activation.

## 4 Discussion

This study examined temporal discounting for self and friend and the associated neural correlates in adolescence. To this end, we examined adolescents’ temporal discounting in task conditions where they had to decide between immediate and delayed rewards for either themselves and their friend, and task conditions where immediate and delayed rewards for self and friend were juxtaposed (Albrecht et al., 2011). The results align with prior studies that exclusively investigated temporal discounting for self, confirming age related decreases in temporal discounting (de Water et al., 2014, Olson et al., 2007, Scheres et al., 2006, Steinberg et al., 2009). We showed that there was an age-related increase in parietal cortex activation for deliberating temporal discounting choices (de Water et al., 2017). Next, we confirmed that participants who more often chose the immediate reward showed higher activity in the subgenual ACC/striatum when making immediate choices (Albrecht et al., 2011). Regarding social decisions, adolescents showed more impulsivity when deliberating immediate rewards for their friend versus delayed rewards for themselves, compared to other task conditions. Finally, the temporal parietal junction (TPJ) and precuneus were exclusively activated for choices in which immediate rewards for self were pitted against larger delayed rewards for friends. These findings confirm that choosing between immediate versus delayed rewards in adolescence depends on the beneficiary of the rewards.

### 4.1 Temporal discounting for self and others in adolescence

The novel contribution of this study was examining neural similarity and differences for personal and social temporal discounting. We expected that adolescents would more often select the delayed reward in decisions involving the friend, as such decisions may be less impacted by sensitivity to rewards for the self and more by mentalizing about the consequences for others (Mahy et al., 2020, Neff and Macaskill, 2021). Interestingly, results showed that adolescents preferred immediate rewards (reflected by a lower AUC) when balancing immediate rewards for their friend and delayed rewards for themselves, compared to other task conditions. This finding shows that participants sacrificed a large, delayed reward for self for an immediate reward for their friend. Notably, this preference for rewards for friends was not observed for the reverse condition. Possibly, foregoing immediate rewards for the self to benefit the friend requires more effort than the reverse (Lockwood et al., 2017). Indeed, we observed that deliberating a delayed reward for the friend instead of an immediate reward for the self was associated with increased activation in the right precuneus and right TPJ. The precuneus and TPJ are social brain regions that have both been implicated in perspective taking and mentalizing (Blakemore, 2008, Crone and Dahl, 2012, Crone and Fuligni, 2020, Van Overwalle and Baetens, 2009). Therefore possibly, considering delayed outcomes for friends when this comes at the expense of personal immediate gratification is associated with an increased need to switch perspectives between self and friend or to effortfully take the perspective of the other (Carter and Huettel, 2013, Crone and Dahl, 2012).

Next, we observed that adolescents did not differentiate in their behavior or neural activation between decisions solely involving the self or the friend. Possibly, adolescents make decisions solely involving the friend similarly as decisions solely involving the self, for example being guided by an assumption that they and their friend are similar and want similar outcomes (Aron et al., 1991). Our results suggest that only when the friend and self are juxtaposed adolescents increasingly consider their friend either in behavior or TPJ and precuneus activation. This suggests that different processes are at play when temporal decisions for self and other are juxtaposed compared to when they are not.

### 4.2 Temporal discounting in adolescence: general patterns

Consistent with prior studies, we observed that older adolescents were less impulsive in the context of temporal discounting than younger adolescents (Christakou et al., 2011, de Water et al., 2014, Ripke et al., 2012, Scheres et al., 2013), which was observed across all task conditions. Also consistent with prior studies, we observed that temporal discounting choices versus control choices elicited activation in various cortical and subcortical brain regions bilaterally, such as the lateral prefrontal cortex, anterior insula, and ventral striatum (de Water et al., 2017, Scheres et al., 2013, Stanger et al., 2013, Steinberg et al., 2009). This suggests that temporal discounting choices in general depend on the interplay of more controlled processes (e.g., reflected in lPFC activation) and emotional processes (e.g., reflected in insular and striatal activation), which is consistent with the neurobiological dual systems model of temporal discounting (Scheres et al., 2013). Interestingly, adolescents showed increased activation in the right inferior parietal lobule (IPL) with age for temporal discounting versus control choices. The IPL has been implicated in a myriad of functions, including attentional control, thinking about the past and future, and social cognition (Igelström and Graziano, 2017). Within the context of temporal discounting, increased IPL activation has been associated with choosing or experiencing a higher value of the delayed reward (Frost and McNaughton, 2017). One other developmental study comparing temporal discounting in adolescents (13 – 15 years) and adults (19 – 50 years) also observed more IPL activity in adults compared to adolescents, which was related to more consistent choices (Ripke et al., 2012). Here, consistency was defined as the degree to which subjects invariably choose the alternative with the higher subjective value over the time course of the task. This measure may reflect how confident adolescents feel about their strategy to select delayed rewards. The authors speculated that the increased maturation of the parietal cortex enables individuals to compare immediate and delayed rewards more precisely. Combined, this suggests that the right IPL plays a role in the maturation of deliberation in adolescents’ decision making.

We also addressed the question whether there were neural regions that correlated with immediate versus delayed choice behavior. Comparing delayed and immediate reward choices on average revealed no significant clusters of activation. Instead, immediate vs. delayed reward choices were dependent on individual differences. We observed that more impulsive adolescents showed higher activation in the subgenual ACC, extending into the left and right ventral striatum, for immediate versus delayed rewards for the self relative to less impulsive adolescents. This is in line with prior studies suggesting that individual differences in reward sensitivity and, specifically, sensitivity to the immediacy of rewards, may contribute to self-oriented temporal discounting (Albrecht et al., 2011, de Water et al., 2017, Ripke et al., 2012, Scheres et al., 2013). Interestingly, similar to de Water et al. (2017), we did not observe age effects in the mPFC, ventral striatum, and DLPFC, even though these regions have previously been suggested to underlie changes in temporal discounting for the self across adolescent development (de Water et al., 2014, Scheres et al., 2013, Van Den Bos et al., 2015). These findings are inconsistent with neurobiological models of adolescent development, which pose that adolescents’ relative impulsivity and temporal discounting are driven by the interplay of heightened reward sensitivity and the protracted development of cognitive control capabilities (Casey et al., 2008, Crone and Dahl, 2012, Galvan et al., 2007, Steinberg et al., 2009). Together, these findings suggest that adolescents’ temporal discounting behavior is more sensitive to individual preferences and brain regions may be recruited depending on the strategic motives or salience of the choice, outcome, and beneficiary (Albrecht et al., 2011, de Water et al., 2017, Van Den Bos et al., 2015). Future studies should therefore test in more detail how temporal discounting is affected by the social consequences of the participants and the partners for whom they are playing. The current study provides the first indication that these choices and neural activity are modulated by the beneficiary of the choices.

Developmental reductions in temporal discounting across adolescence may be better explained by changes in future orientation, which may be supported by specific changes in functional and anatomical dlPFC-striatal connectivity, than by heightened sensitivity to immediate rewards (Van Den Bos et al., 2015). Alternative explanations for the inconsistent findings regarding the contribution of striatal and cortical regions to the decrease in temporal discounting in adolescents may relate to the type of discounting assessed, the exact paradigm that is being used, or the imaging modalities employed (e.g., functional connectivity vs. general task-based fMRI), which should be addressed in future research. For example, some studies demonstrated social context dependency, such that striatal activity only contributes to age-related changes in temporal discounting in adolescence when decisions are being observed by peers (Chein et al., 2011; Van Den Bos et al., 2015).

Overall, our results suggest that adolescents’ temporal discounting decisions for the self are influenced by individual differences in reward sensitivity (i.e., subgenual ACC/bilateral ventral striatum activity), whereas selecting immediate and delayed rewards for friends when these are juxtaposed with rewards for the self may be easier or more difficult depending on whether the reward for friend is immediate or delayed, respectively. Selecting the reward for the friend may in the first case reflect prosocial tendencies, whereas it may be effortful and requires perspective taking in the second case. As such, choices that involve both a temporal and social component are likely to not only be impacted by reward sensitivity and cognitive control, but also by social cognition and prosocial motives (Crone et al., 2022). Indeed, prior research has shown that individuals are less generous towards others when payments are delayed within the context of zero-sum games, such as Dictator Games (Kovarik, 2009), suggesting that prosocial and temporal processes are often intertwined.

### 4.3 Limitations and future directions

This study has several limitations and provides directions for future research. First, the current study cross-sectionally examined age-related differences in temporal discounting for self and friends. Future studies should focus on mapping within-person development across longitudinal studies. Second, we here focused on temporal discounting for self versus friend, but future studies could work out the extent to which social distance shapes temporal choices for self and others by including other targets, such as unfamiliar others and family members. Third, some participants could not be included in all analyses, because they showed too little variability in their temporal discounting choices. This can be resolved by using designs which adjust the immediate reward amount to participants’ decisions (Christakou et al., 2011), or which use participant-specific choice sets based on a pre-test outside the MRI scanner (Van Den Bos et al., 2015). Fourth, although we, like other studies, found evidence that adolescents’ sensitivity to immediate rewards drives their impatience in the condition solely involving the self (Albrecht et al., 2011, McClure et al., 2004), there also have been studies which have failed to find this effect (Van Den Bos et al., 2015), or that suggest a regulatory role for the ventral striatum such that it drives delayed reward choices in highly impatient individuals (de Water et al., 2017). Possibly, the exact role of the ventral striatum in decision making depends on the emotional and social saliency of the task (Telzer, 2016). Fifth, although we incorporated a social component in our temporal discounting paradigm by having outcomes for both the self and a friend, real-life temporal discounting choices are more complicated and likely to be influenced by other social processes, such as peer presence, group dynamics, and perceived social norms. Future research should aim to investigate their influence on temporal discounting choices for self and others. Finally, we used AUC as a measure of temporal discounting because this warrants comparability with other studies using the same version of the temporal discounting task in adolescents (e.g., (de Water et al., 2017). However, it should be noted that there are also other ways to calculate temporal discounting, such as log *k* which may make better use of input from relatively shorter delays (Yoon et al., 2017).

## 5 Conclusion

The current study expands our understanding of temporal discounting behavior across adolescence. By extending existing paradigms with conditions that examined immediate versus delayed reward choice for either self or friend, and conditions that juxtaposed such decisions for self and friend, we demonstrated that adolescents’ temporal decisions are influenced by the social context. Specifically, we have demonstrated that adolescents forego immediate rewards for self to benefit their friends, which challenges existing stereotypes about adolescents being self-centered or generally unable to delay gratification. As such, this study suggests that AUC is influenced by motivational factors and that adolescents’ impulsivity may be strengthened when this matches their social goals (Crone and Fuligni, 2020). Our study suggests that temporal decisions involving the self rely on different neural processes compared to temporal decisions juxtaposing outcomes for self and other. First, immediate reward choices for the self were correlated with activity in subgenual ACC/ventral striatum activity, possibly reflecting greater subjective enjoyment, specifically for impulsive individuals. Second, making temporal choices for friends was associated with increased TPJ and precuneus activation, possibly reflecting mentalizing processes that interact with decision-making (Albrecht et al., 2011, Crone et al., 2022). Finally, the right IPL may contribute to the maturation of adolescents’ temporal choices which may signal increased consistency and confidence of choices (Ripke et al., 2012). Together, these findings highlight behavioral and neural mechanisms underlying adolescents’ temporal decision making for self and friends, which may impact the balance between adolescents’ personal and interpersonal goal achievement, and thus social adjustment, later in life.

## Funding

This work was supported by an innovative ideas grant of the 10.13039/501100000781European Research Council (ERC CoG PROSOCIAL 681632 to E.A.C.).

We would like to thank all participants for their participation, and Anna van Steenbergen, Kayla Green, Cevdet Acarsoy, Suzanne Splinter, Bram Zwanenburg, Maaike Verburg, Esther de Wit, and Zaida Amorij for their help with data collection.

## Declaration of Competing Interest

The authors declare that they have no known competing financial interests or personal relationships that could have appeared to influence the work reported in this paper.

## Data Availability

Data will be made available upon request through the Data Repository of Erasmus University Rotterdam after acceptance of the current article.
