# Supplementary material for: Temporal discounting for self and friends in adolescence: A fMRI study
Source: Dev Cogn Neurosci. 2023 Jan 27;60:101204. doi: 10.1016/j.dcn.2023.101204 (PMC9918426; doi:10.1016/j.dcn.2023.101204)
Supplement: Supplementary file 1 — Supplementary material. [file mmc1.docx]

**Supplement 1. Combinations of combination of task conditions, immediate rewards, and delays**

**Supplement 2: Behavioral effects of delays in days and immediate reward magnitude**

**Effect of delay in days on temporal discounting in the four task conditions.** Adding delay in days as a within-subjects factor to the RM ANOVA assessing task condition effects revealed both a main effect of delay, *F*(1.84, 175.12) = 96.03, *p* < .001, *η*²_p_ = .50, as well as an interaction between delay and task condition, *F*(9.08, 862.66) = 10.22, *p* < .001, *η*²_p_ = .10. Follow-up analyses examining repeated Bonferroni-corrected pairwise comparisons for each of the task conditions in separate repeated measures ANOVAs revealed that the subjective value of the delayed reward decreased monotonically with increasing delay in the ‘Self Immediate – Self Delay’ and ‘Friend Immediate – Self Delay’ conditions, *p’s* ≤ .010. The other task conditions largely showed the same pattern, but there were no differences between a delay of 2 and 14 days and 90 and 180 days in the ‘Friend Immediate – Friend Delay’ conditions, and between 14 and 30 days in the ‘Self Immediate – Friend Delay’ condition, see Figure S1. Adding linear and quadratic age to the analysis revealed no interactive effects of delay in days and age, suggesting that the different delays were processed similarly across adolescence.

**Effect of immediate reward magnitude on temporal discounting in the four task conditions.** Adding reward magnitude as a within-subjects factor to the RM ANOVA assessing task conditions effects revealed a main effect of reward magnitude, *F*(1.77, 168.29) = 87.48, *p* < .001, *η*²_p_ = .48, as well as an interaction between reward magnitude and task condition, *F*(6.40, 607.83) = 20.89, *p* < .001, *η*²_p_ = .18. Follow-up analyses examining repeated Bonferroni-corrected pairwise comparisons for each of the task conditions in separate repeated measures ANOVAs suggested that on average, the subjective value of the delayed reward decreased with increasing immediate reward value, but the exact pattern of the SV of immediate reward magnitude differed per condition, see Figure S2A. Adding linear age to the analysis revealed an interaction effect between linear age and immediate reward magnitude, *F*(1.80, 169.61) = 3.52, *p* = .036, *η*²_p_ = .04. Follow-up analyses averaged over task conditions showed that age was positively related to the subjective value of the delayed reward for an immediate reward value of 2 (*B* = .32, *p* < .001), 4 (*B* = .29, *p* < .001), and 6 (*B* = .26, *p* =.004), but not 8, *p* = .074. Adding quadratic age to the analysis (which also included linear age as a predictor) revealed an interaction effect between quadratic age and immediate reward magnitude (and the linear effect of age disappeared), *F*(1.84, 178.28) = 3.97, *p* = .024, *η*²_p_ = .04. Follow-up analyses averaged over task conditions revealed a difference between reward magnitudes 2 and 4 on the one hand, and 8 on the other hand, *p*’s < .05. As can be seen in Figure S2b, relative to the SV’s of the immediate reward magnitudes of 2 and 4, the SV of the immediate reward magnitude of 8 showed a more negative quadratic trajectory (i.e., a mid-adolescent dip).


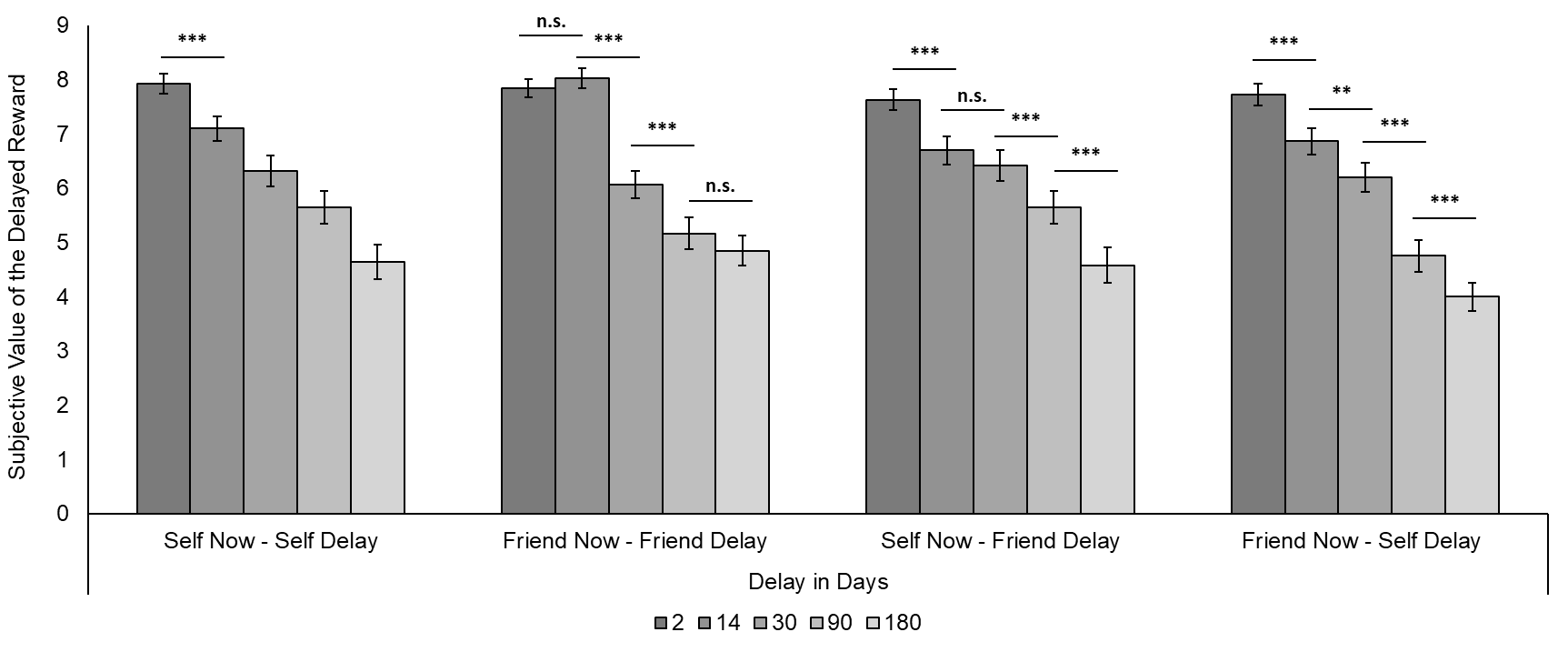


Figure S1. The subjective value (SV) of the delayed reward for each of the delays for the four task conditions. The subjective value of the delayed reward decreased monotonically with increasing delays, except for some comparisons in the ‘Friend Immediate – Friend Delay’ and ‘Friend Immediate – Self Delay’ conditions. ** denotes p-values ≤ .010, *** denotes p-values ≤ .001.


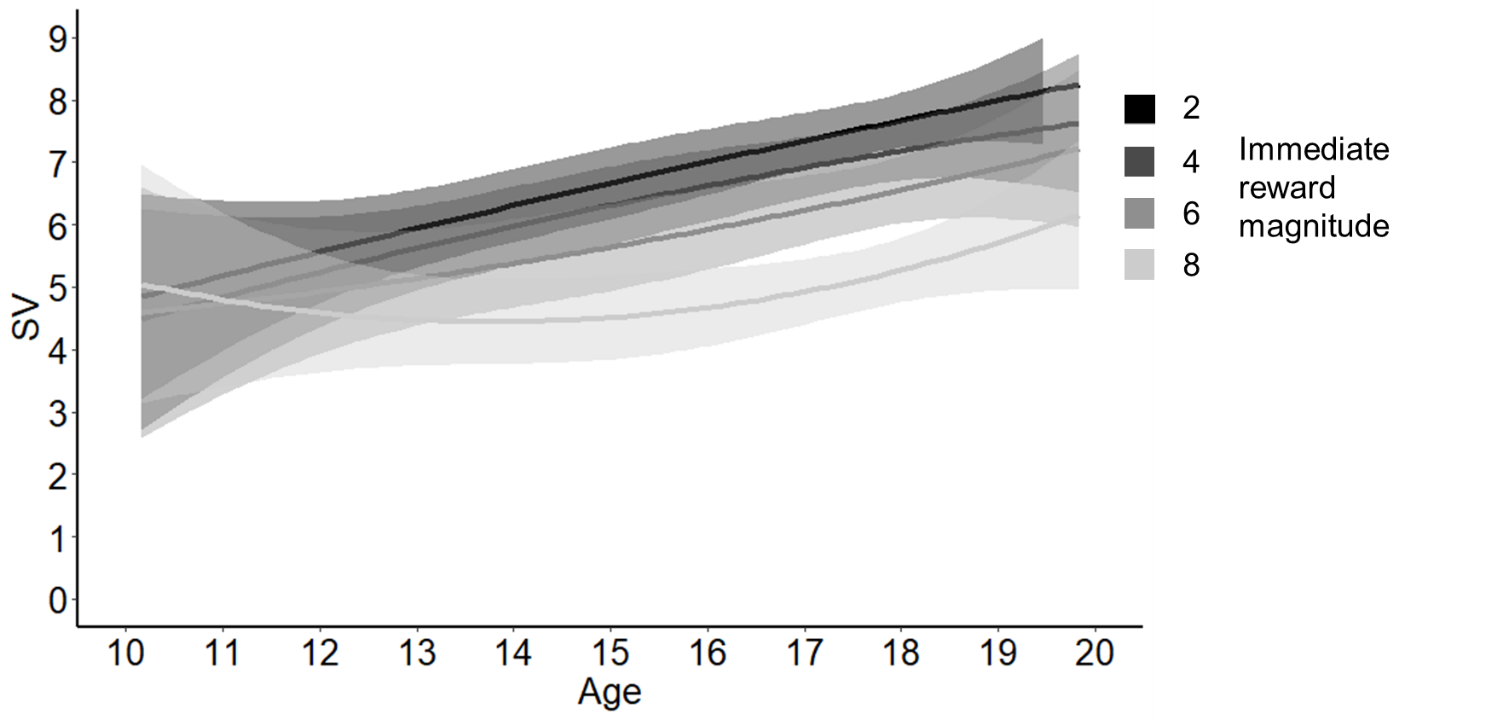

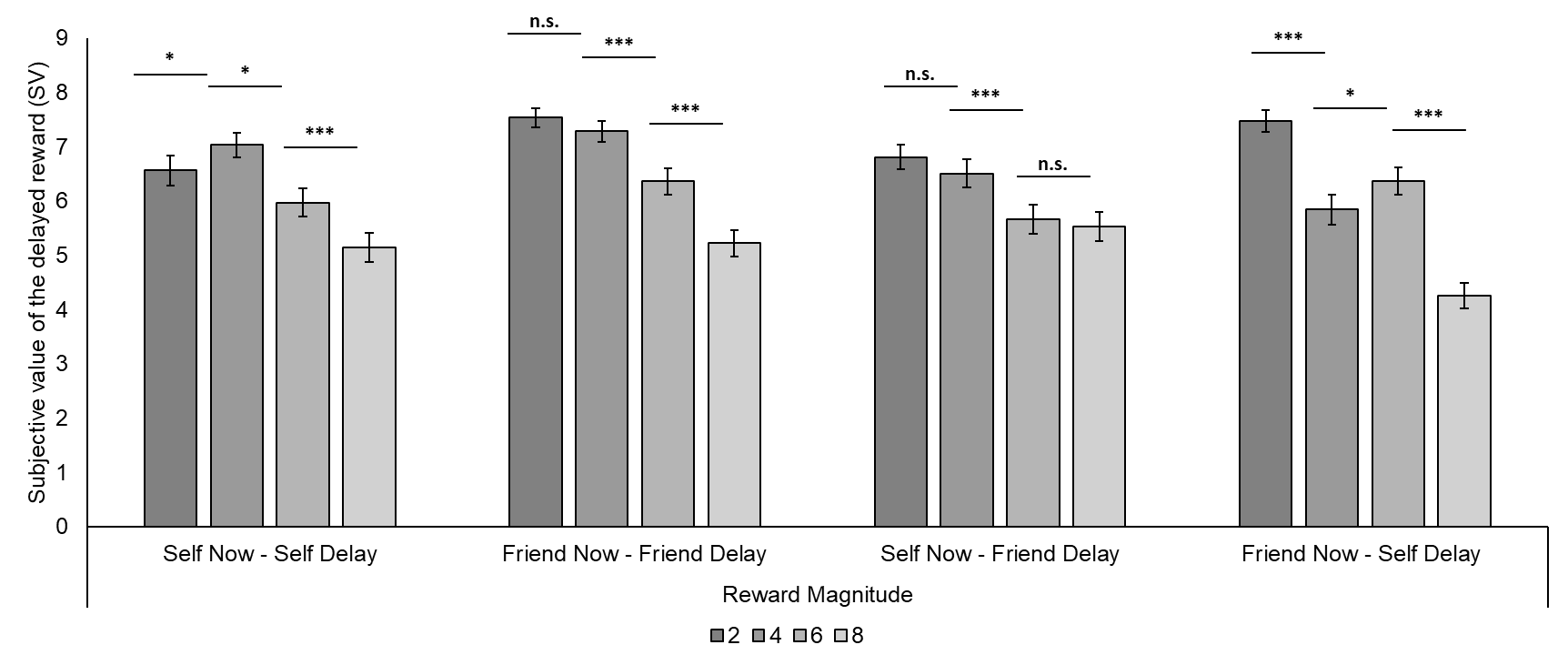


Figure S2B. Association between age and the subjective value of the delayed reward for each of the four immediate reward values.

Figure S2A. The subjective value (SV) of the delayed reward for each immediate reward magnitude for the four task conditions. * denotes p-values ≤ .050, ** denotes p-values ≤ .010, *** denotes p-values ≤ .001.

| *Measures* | *Average values* |
| --- | --- |
| Means (SD) | .089 (.09) |
| Minimum | .005 |
| Maximum | .088 |
| Number of small spikes (.5-3mm) | 7.89 (1.35% of total volumes) |
|  | *Number* |
| Participants with 0 spikes (.5-3mm) | 37 |
| Participants with small spikes (.5-3mm) | 59 |

**Supplement 3. Movement information**

**Supplementary Table S3.1:** Movement information after exclusion of participants with >.3mm movement


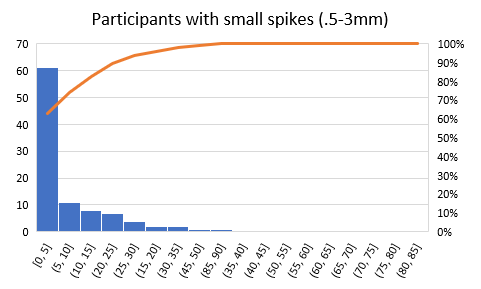


**Supplementary Figure S3.1:** Number of participants with small spikes (.5-3mm). The x-axis shows categories reflecting the number of small spikes. The y-axis reflects the number of participants within each category.

| PPN | Mean | stdev | min | max | Nr of spikes >.5 mm |
| --- | --- | --- | --- | --- | --- |
| BL2P065 | 0.075538 | 0.082455 | 0.00438 | 1.040033 | 4 |
| BL2P067 | 0.156443 | 0.171568 | 0.004609 | 1.440395 | 30 |
| BL2P068 | 0.065917 | 0.056008 | 0.003173 | 0.668539 | 2 |
| BL2P070 | 0.152065 | 0.141359 | 0.005225 | 1.620346 | 13 |
| BL2P071 | 0.105551 | 0.115311 | 0.006051 | 1.166376 | 9 |
| BL2P072 | 0.049233 | 0.028942 | 0.006502 | 0.37551 | 0 |
| BL2P074 | 0.16841 | 0.144839 | 0.002334 | 0.9647 | 22 |
| BL2P075 | 0.055867 | 0.034318 | 0.003444 | 0.30976 | 0 |
| BL2P077 | 0.075496 | 0.091696 | 0.001358 | 0.857369 | 6 |
| BL2P079 | 0.067365 | 0.042283 | 0.004897 | 0.498558 | 0 |
| BL2P081 | 0.04677 | 0.031587 | 0.003476 | 0.31787 | 0 |
| BL2P082 | 0.051851 | 0.031508 | 0.003432 | 0.194638 | 0 |
| BL2P083 | 0.083945 | 0.126054 | 0.00458 | 1.635819 | 11 |
| BL2P084 | 0.068153 | 0.067657 | 0.005334 | 0.790424 | 1 |
| BL2P085 | 0.089967 | 0.083051 | 0.00875 | 0.668189 | 3 |
| BL2P086 | 0.058258 | 0.049088 | 0.004898 | 0.584283 | 1 |
| BL2P088 | 0.121366 | 0.167681 | 0.006261 | 1.932651 | 22 |
| BL2P092 | 0.070467 | 0.041727 | 0.009564 | 0.241602 | 0 |
| BL2P093 | 0.048797 | 0.032834 | 0.00324 | 0.290135 | 0 |
| BL2P094 | 0.06775 | 0.07444 | 0.003437 | 0.662584 | 5 |
| BL2P095 | 0.04806 | 0.036311 | 0.004783 | 0.319072 | 0 |
| BL2P096 | 0.073977 | 0.048592 | 0.006837 | 0.418301 | 0 |
| BL2P097 | 0.068509 | 0.060179 | 0.004304 | 0.599577 | 2 |
| BL2P098 | 0.056625 | 0.039525 | 0.006016 | 0.335726 | 0 |
| BL2P099 | 0.044154 | 0.031178 | 0.005118 | 0.343744 | 0 |
| BL2P100 | 0.144814 | 0.165709 | 0.005319 | 1.635161 | 25 |
| BL2P101 | 0.15416 | 0.173645 | 0.008378 | 1.562714 | 23 |
| BL2P102 | 0.128798 | 0.188449 | 0.002014 | 1.864652 | 18 |
| BL2P105 | 0.073758 | 0.061043 | 0.004393 | 0.539388 | 0 |
| BL2P106 | 0.094479 | 0.101975 | 0.005618 | 0.745738 | 8 |
| BL2P109 | 0.040513 | 0.025227 | 0.002011 | 0.197887 | 0 |
| BL2P111 | 0.075072 | 0.055961 | 0.005302 | 0.466885 | 0 |
| BL2P112 | 0.095989 | 0.123288 | 0.005143 | 1.382417 | 10 |
| BL2P113 | 0.129105 | 0.128808 | 0.007352 | 1.13098 | 13 |
| BL2P116 | 0.072749 | 0.048248 | 0.00276 | 0.469067 | 0 |
| BL2P117 | 0.121031 | 0.127127 | 0.00705 | 1.000348 | 14 |
| BL2P121 | 0.05206 | 0.047312 | 0.002966 | 0.446651 | 0 |
| BL2P122 | 0.155648 | 0.215123 | 0.010146 | 2.167455 | 27 |
| BL2P124 | 0.080326 | 0.060315 | 0.002306 | 0.453261 | 0 |
| BL2P125 | 0.052762 | 0.04444 | 0.00198 | 0.366445 | 0 |
| BL2P127 | 0.113905 | 0.190362 | 0.004551 | 2.247658 | 15 |
| BL2P128 | 0.052043 | 0.027748 | 0.003095 | 0.162317 | 0 |
| BL2P130 | 0.047209 | 0.029275 | 0.00372 | 0.290358 | 0 |
| BL2P131 | 0.13179 | 0.194122 | 0.003507 | 2.494242 | 27 |
| BL2P132 | 0.086779 | 0.102963 | 0.003502 | 1.302684 | 3 |
| BL2P133 | 0.065117 | 0.068775 | 0.001909 | 0.750479 | 3 |
| BL2P134 | 0.091272 | 0.086842 | 0.003934 | 0.964557 | 4 |
| BL2P137 | 0.09938 | 0.097923 | 0.002474 | 1.201832 | 6 |
| BL2P138 | 0.057258 | 0.04363 | 0.005336 | 0.418426 | 0 |
| BL2P139 | 0.122419 | 0.205268 | 0.002481 | 2.727965 | 22 |
| BL2P141 | 0.185635 | 0.210971 | 0.011408 | 2.018473 | 33 |
| BL2P142 | 0.142344 | 0.191637 | 0.002836 | 1.86649 | 32 |
| BL2P144 | 0.097372 | 0.082874 | 0.008629 | 0.851227 | 4 |
| BL2P148 | 0.064018 | 0.044139 | 0.005689 | 0.56638 | 1 |
| BL2P149 | 0.062764 | 0.034515 | 0.007528 | 0.285308 | 0 |
| BL2P153 | 0.058479 | 0.048543 | 0.004048 | 0.61083 | 2 |
| BL2P154 | 0.096764 | 0.117417 | 0.001333 | 0.946264 | 12 |
| BL2P155 | 0.112991 | 0.163938 | 0.003842 | 2.399158 | 15 |
| BL2P157 | 0.129043 | 0.098128 | 0.012932 | 0.763968 | 6 |
| BL2P160 | 0.096576 | 0.091626 | 0.010263 | 0.982715 | 4 |
| BL2P161 | 0.275313 | 0.318499 | 0.013311 | 2.750901 | 87 |
| BL2P162 | 0.074627 | 0.072996 | 0.005959 | 0.731556 | 3 |
| BL2P163 | 0.069127 | 0.052518 | 0.002824 | 0.47521 | 0 |
| BL2P167 | 0.105071 | 0.113367 | 0.004291 | 0.916393 | 11 |
| BL2P168 | 0.107834 | 0.147662 | 0.004331 | 2.012096 | 10 |
| BL2P169 | 0.110502 | 0.087812 | 0.008152 | 0.683609 | 6 |
| BL2P170 | 0.051152 | 0.028048 | 0.005135 | 0.168938 | 0 |
| BL2P172 | 0.063134 | 0.036631 | 0.004304 | 0.407922 | 0 |
| BL2P173 | 0.084453 | 0.090398 | 0.003819 | 0.999571 | 7 |
| BL2P174 | 0.053754 | 0.040373 | 0.006695 | 0.520552 | 1 |
| BL2P176 | 0.053774 | 0.031609 | 0.001767 | 0.244532 | 0 |
| BL2P177 | 0.052043 | 0.027748 | 0.003095 | 0.162317 | 0 |
| BL2P178 | 0.108286 | 0.081675 | 0.006997 | 0.579818 | 3 |
| BL2P179 | 0.06309 | 0.045198 | 0.008866 | 0.311517 | 0 |
| BL2P180 | 0.123533 | 0.179644 | 0.002538 | 1.458461 | 25 |
| BL2P182 | 0.068613 | 0.067378 | 0.006114 | 0.845575 | 2 |
| BL2P183 | 0.166002 | 0.283808 | 0.006392 | 2.479941 | 47 |
| BL2P185 | 0.102417 | 0.071529 | 0.006424 | 0.374169 | 0 |
| BL2P186 | 0.073616 | 0.050746 | 0.007535 | 0.494966 | 0 |
| BL2P187 | 0.070263 | 0.039355 | 0.002053 | 0.288386 | 0 |
| BL2P189 | 0.088159 | 0.094834 | 0.002618 | 0.797948 | 4 |
| BL2P190 | 0.082193 | 0.069863 | 0.003922 | 0.538227 | 2 |
| BL2P191 | 0.049863 | 0.030165 | 0.004484 | 0.221802 | 0 |
| BL2P192 | 0.100207 | 0.097568 | 0.00556 | 0.811261 | 9 |
| BL2P193 | 0.127495 | 0.138887 | 0.004768 | 1.198526 | 20 |
| BL2P194 | 0.042556 | 0.0233 | 0.003561 | 0.146468 | 0 |
| BL2P198 | 0.068356 | 0.058215 | 0.005861 | 0.51157 | 1 |
| BL2P199 | 0.166206 | 0.230315 | 0.005598 | 2.139739 | 29 |
| BL2P203 | 0.080637 | 0.062621 | 0.002441 | 0.779854 | 1 |
| BL2P204 | 0.102225 | 0.090474 | 0.002881 | 0.81219 | 5 |
| BL2P205 | 0.069157 | 0.050352 | 0.004583 | 0.500733 | 1 |
| BL2P211 | 0.045343 | 0.030691 | 0.003506 | 0.338458 | 0 |
| BL2P212 | 0.041356 | 0.024295 | 0.002645 | 0.17902 | 0 |
| BL2P213 | 0.070743 | 0.039768 | 0.002618 | 0.338262 | 0 |
| BL2P214 | 0.148231 | 0.148814 | 0.011628 | 1.124666 | 25 |
| BL2P215 | 0.056883 | 0.047994 | 0.004113 | 0.449494 | 0 |
| Average | 0.089283 | 0.089924 | 0.004991 | 0.878659 | 7.885417 |

**Supplementary Table S3.2.** Specific movement information for the 35 participants who displayed small motion spikes (.9-3mm).

**Data access**

Raw data for each participant can be provided by the first author upon request.

**Supplement 4. Additional neuroimaging analyses**

**4.1. Control versus Temporal Discounting.**

The contrast ‘Control vs Temporal Discounting’ (FWE and FDR corrected, *p* <.050; *k* ≥ 13; *N* = 84) revealed increased activation in the mPFC, left lateral PFC, bilateral TPJ, right insula, and right precuneus, amongst others, *t*’s ≥ 5.88, p's ≤ 0.001, see Table S4.1 and Figure S4.


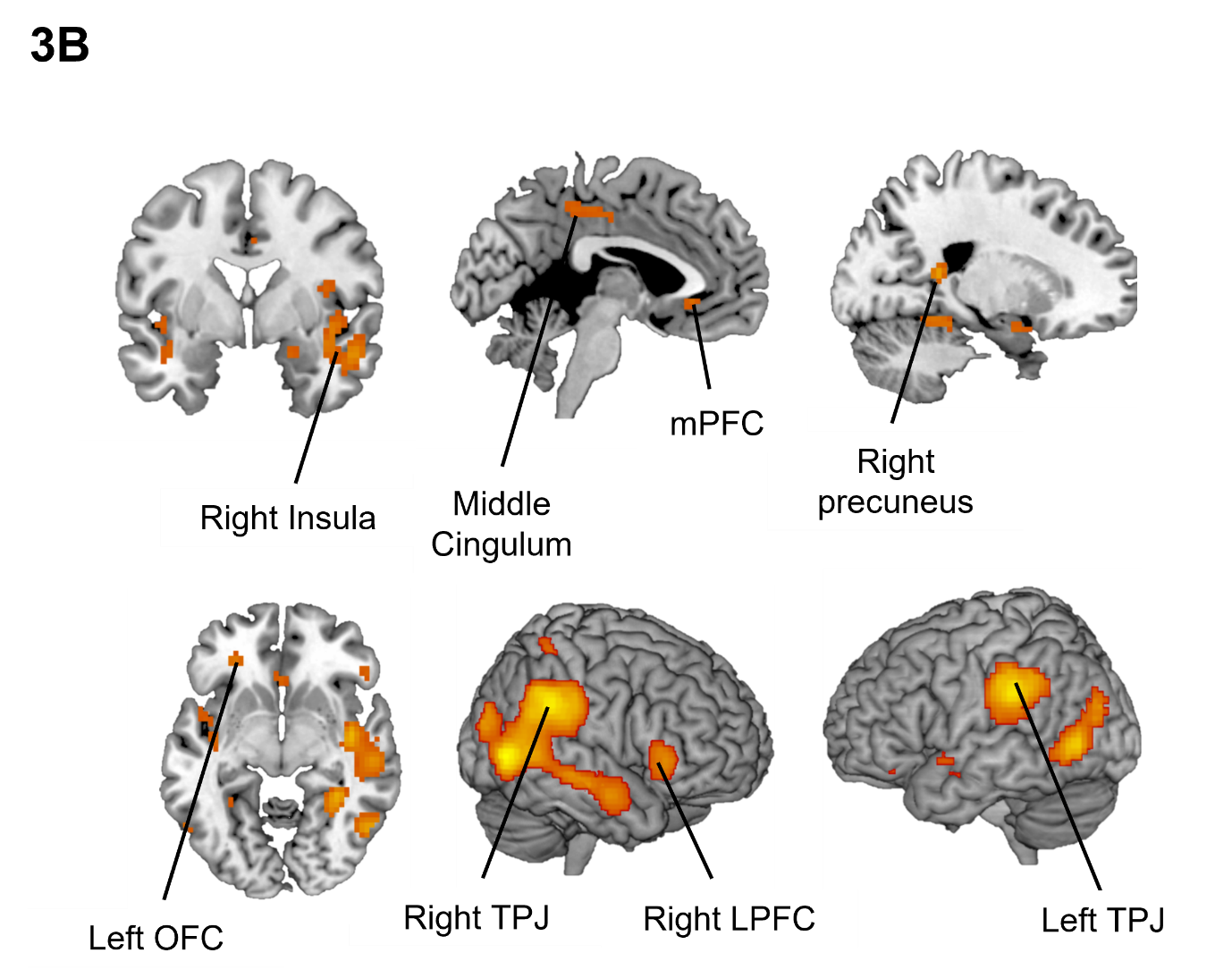


**‘Control vs. Temporal Discounting’**

FWE corrected, FDR corrected, *p* < .05, *k* ≥ 13

Figure S4. Brain areas that showed increased activation for the t-test ‘Control vs. Temporal Discounting’. Results are displayed FWE and FDR cluster correction of p < .050.

| Table S4.1 |  | | | | | |  |  |
| --- | --- | --- | --- | --- | --- | --- | --- | --- |
| MNI coordinates of local maxima activated for the t-test for control versus TD. Results were calculated using FWE and FDR cluster correction (*p ≤* .050). | | | | | | | | |
| Area of activation | MNI Coordinates | |  | | | Test statistic | | Cluster Size |
|  | x | y | | z |  | *t* | |  |
| *T-test Control vs. Delay choices* |  |  | |  |  |  | |  |
| Right Temporal Middle (i.e., right TPJ within this cluster) | 57 | -61 | | 1 |  | 14.59 | | 1445 |
| Left SupraMarginal (i.e., left TPJ within this cluster) | -63 | -34 | | 31 |  | 13.21 | | 256 |
| Left Temporal Middle | -54 | -70 | | 1 |  | 11.62 | | 174 |
| Right Fusiform | 33 | -43 | | -11 |  | 10.08 | | 183 |
| Right Precuneus | 24 | -46 | | 10 |  | 9.23 | | 29 |
| Left Inferior Orbital (i.e., OFC) | -27 | 35 | | -11 |  | 8.60 | | 29 |
| Right Frontal Inferior Triangularis (i.e., Right LPFC) | 51 | 32 | | 1 |  | 7.93 | | 55 |
| Right Middle Cingulum | 9 | -25 | | 43 |  | 7.90 | | 213 |
| Right Postcentral | 33 | -43 | | 58 |  | 6.65 | | 31 |
| Left Anterior Cingulum (i.e., mPFC) | 0 | 26 | | -5 |  | 6.43 | | 16 |
| Right Insula | 39 | 2 | | 13 |  | 5.96 | | 13 |
| Left Temporal Superior | -39 | -10 | | -8 |  | 5.91 | | 54 |
| Left Fusiform | -24 | -46 | | -14 |  | 5.88 | | 22 |

**4.2. Multiple Regression Analysis with Quadratic Age for ‘Temporal discounting vs. Control’.**

| Table S4.2 |  | | | | | |  |  |
| --- | --- | --- | --- | --- | --- | --- | --- | --- |
| MNI coordinates of local maxima activated for the multiple regression comparing temporal discounting to control trials depending on quadratic age. Results were calculated using a primary voxel-wise threshold of *p* < .001 (uncorrected), with a cluster corrected threshold of *p* < .050 FDR corrected. | | | | | | | | |
| Area of activation | MNI Coordinates | |  | | | Test statistic | | Cluster Size |
|  | x | y | | z |  | *t* | |  |
| *Multiple regression for ‘Temporal Discounting vs. Control Trials’  – clusters that regressed with quadratic age* |  |  | |  |  |  | |  |
|  |  |  | |  |  |  | |  |
| Right Occipital Superior | 33 | -70 | | 43 |  | 3.84 | | 83 |

A whole brain multiple regression analysis for the comparison ‘Temporal Discounting vs. Control’ with quadratic age (*N* = 84) revealed a cluster of activation in the superior occipital lobe, see Table S4.2.

Note: Names were based on the aal toolbox in SPM. For functional regions discussed throughout the paper, both the aal label and functional label (between brackets) are displayed. See [*https://neurovault.org/collections/FZTVRFUL/*](https://neurovault.org/collections/FZTVRFUL/) for a full, unthresholded overview of activation.
